# Decision making for anti-VEGF inhibitor continuation: dip stick? or urine protein/creatinine ratio? (VERSiON UP study)

**DOI:** 10.1186/s12885-022-09611-3

**Published:** 2022-05-07

**Authors:** Michio Nakamura, Taro Funakoshi, Shigeki Kataoka, Takahiro Horimatsu, Yoshitaka Nishikawa, Takeshi Matsubara, Takuro Mizukami, Tomoyuki Goto, Kenji Tsuchihashi, Eishi Baba, Takehiko Tsumura, Yoshiaki Mihara, Tetsuya Hamaguchi, Motoko Yanagita, Manabu Muto

**Affiliations:** 1 Department of Gastroenterology, Sapporo City General Hospital, 1-1, Kita 11-jo Nishi 13-chome, Chuo-ku, Sapporo, 060-8648 Japan; 2 Department of Clinical Oncology, Kyoto University Hospital, 54 Shogoin-kawahara-cho, Sakyo-ku, Kyoto, 606-8507 Japan; 3 Department of Health Informatics, Kyoto University School of Public Health, Yoshida-Konoe-cho, Sakyo-ku, Kyoto, 606-8501 Japan; 4 Department of Nephrology, Graduate School of Medicine, Kyoto University, 54 Shogoin-Kawahara-cho, Sakyo-ku, Kyoto, 606-8507 Japan; 5 Department of Clinical Oncology, St. Marianna University School of Medicine, 2-16-1 Sugao, Miyamae-ku, Kawasaki, 216-8511 Japan; 6 Department of Gastroenterology, Shiga General Hospital, 4-30, Moriyama-5-chome, Moriyama, 524-8524 Japan; 7 Department of Hematology, Oncology & Cardiovascular Medicine, Kyushu University Hospital, 3-1-1 Maidashi, Higashi-ku, Fukuoka, 812-8582 Japan; 8 Department of Oncology and Social Medicine, Graduate School of Medical Sciences, Kyushu University, 3-1-1 Maidashi, Higashi-ku, Fukuoka, 812-8582 Japan; 9 Department of Medical Oncology, Osaka Red Cross Hospital, 5-30 Fudegasakicho, Tennoji-ku, Osaka, 543-8555 Japan; 10 Department of Medical Oncology, Saitama Medical University International Medical Center, 1397-1, Yamane, Hidaka, 350-1298 Japan

**Keywords:** Anti-vascular endothelial growth factor therapy, Gastrointestinal cancer, Proteinuria, Urine protein/creatinine ratio

## Abstract

**Background:**

Monitoring proteinuria is important for the management of patients with cancer treated with anti-vascular endothelial growth factor (VEGF) or anti-VEGF receptor (VEGFR) inhibitors (VEGF/Ri). Here we investigated the difference between the urine protein/creatinine ratio (UPCR) and a qualitative value test (QV) on the decision making of treatment continuation and the usefulness of UPCR testing in patients with gastrointestinal cancer treated with anti-VEGF/Ri.

**Methods:**

From January 2017 to December 2018, a survey was conducted based on the medical records of patients with gastrointestinal cancer with a QV of ≥2+ during the use of anti-VEGF/Ri at seven Japanese institutions participating in the Onco-nephrology Consortium. The primary endpoint was the ratio of the worst UPCR < 2.0 (low UPCR) in cases with a QV2+ at the point of the first proteinuria onset. The secondary endpoints were a comparison of low UPCR and worst UPCR ≥2.0 (high UPCR), the concordance rate between UPCR and QV in the Common Terminology Criteria for Adverse Events (CTCAE) grading, and the differences in the decision making for anti-VEGF/Ri continuation.

**Results:**

Among the 71 patients enrolled, the proportion of low UPCR in onset QV2+ (*n* = 53) was 66% (*n* = 35). In a comparison between low (*n* = 36) and high UPCR cases (*n* = 24), body weight (*P* = 0.036), onset QV status (*P* = 0.0134), and worst QV status (*P* < 0.0001) were significantly associated with UPCR levels. The concordance rate for CTCAE Grade 2 of both the QV and UPCR was 83%. Regarding the judgment of anti-VEGF/Ri continuation, treatment was continued in 42.4% of cases when the QV became 3+, whereas only 25% continued treatment when the UPCR value became high.

**Conclusion:**

Urine dipstick test results may overestimate proteinuria, and the UPCR result tended to be more critical than the QV when deciding the treatment policy.

**Trial registration:**

This study is a multiple institutional retrospectively registered observational trial. Clinical Trial number: University Hospital Medical Information Network (UMIN) Clinical Trials Registry (protocol ID UMIN000042545).

**Supplementary Information:**

The online version contains supplementary material available at 10.1186/s12885-022-09611-3.

## Background

Proteinuria is one of the most significant adverse events in managing patients with gastrointestinal cancer treated with anti-vascular endothelial growth factor (VEGF) or anti-VEGF receptor (VEGFR) inhibitors (VEGF/Ri), such as bevacizumab (BV), ramucirumab (Ram), and aflibercept. The incidence of proteinuria varies by study, but it is reported to be approximately 4–65%, including minor cases [1–3]. Although serious cases are relatively rare, severe nephrotic syndrome can occur, and appropriate screening for early detection of severe cases is essential. The gold standard for proteinuria evaluation is a 24-h urine storage test; however, this burdens the patient due to the need for overnight urine collection. Therefore, the qualitative value test (QV) using urine dipstick and the single urine protein/creatinine ratio (UPCR) is widely used in clinical practice [4]. Although the qualitative value test has the advantage of being cheap and convenient, for example, it is not suitable for examining the cutoff value by mathematical processing. Nakamura et al. reported that the UPCR measurement was appropriate for evaluating critical proteinuria in patients with advanced renal cell carcinoma receiving molecular targeted therapy such as tyrosine kinase inhibitors (TKIs) and mammalian target of rapamycin (mTOR) inhibitors, which have antiangiogenic activity [5]. However, it is unknown whether the QV and UPCR are practical for decision making of treatment continuation. Furthermore, regarding the use of the National Cancer Institute Common Terminology Criteria for Adverse Events (CTCAE) grading, the results of the UPCR and QV may not always match, and it may not be easy to decide whether to continue treatment in the actual clinical setting. In the current study, we investigated the difference between the UPCR and QV on the decision making of treatment continuation and the usefulness of UPCR testing for evaluating critical proteinuria in patients with gastrointestinal cancer treated with anti-VEGF/Ri.

## Methods

### Study design

This was a multi-institutional retrospective observation study conducted at seven institutions in Japan, which participated in the Onco-Nephrology consortium. As we extracted the data from the patients’ medical records, neither the patients nor the public were involved in the design, recruitment, or conduct of the study. All living participants signed an informed consent form allowing their data to be used in this study. We announced that the opportunity to opt-out is always available to all participants via a website and posters on hospital walls. The protocol was performed according to the Declaration of Helsinki, the Japanese Ethical Guidelines on Clinical Research, and the Ethical Guidelines for Clinical Studies, and was registered with the University Hospital Medical Information Network (UMIN) Clinical Trials Registry (protocol ID UMIN000042545). Protocol approval was obtained from Kyoto University Graduate School and Faculty of Medicine, Ethics Committee (Approval number and date: R1498, 11/June/2019) and the clinical research ethics review board of all participating institutions.

### Patients

We investigated all patients with gastric and colorectal cancer patients with a QV of ≥2+ during the use of anti-VEGF/Ri between January 2017 and December 2018. The cutoff date for follow-up was October 30, 2019. All clinical data, including patient background factors (e.g., age, sex, body weight, Eastern Cooperative Oncology Group Performance Status Scale [ECOG PS], medical history, complications, comorbidities), primary cancer site, histological subtypes, information on anti-VEGF/Ri (e.g., dose, administration period, treatment continuation), QV status at the point of the first observation of QV (onset QV), and worst QV status, and each UPCR value, were collected by reviewing the patients’ electronic medical records.

### Outcome assessment

The primary objective of this study was to investigate the proportion of cases in which the worst UPCR value was less than 2 (low UPCR) in the cases with an onset QV of 2+ while using an anti-angiogenesis inhibitor. We subsequently investigated how the decision to continue anti-VEGF/Ri treatment based on QV status or UPCR value was made. We also evaluated the comparison of low UPCR and UPCR worst value ≥2.0 (high UPCR) and the risk factors for high UPCR associated with several patient background factors, including age, sex, body weight, ECOG PS, primary cancer site, histological subtypes, used anti-VEGF/Ri, RAS inhibitor usage, prior anti-VEGF/Ri usage, onset QV, and worst QV status.

### Decision making for treatment continuation

The evaluation of the decision making for anti-VEGF/Ri continuation based on changes in QV was performed when the QV became 2+ for the first time, and when it deteriorated from 2+ to 3+. Similarly, regarding the evaluation for the decision making based on changes in the UPCR value, in the low UPCR cases, the assessment was performed when the worst value was recorded. In the high UPCR cases, the evaluation was performed when the UPCR value first became ≥2.0. The decisions were defined by dividing them into three items as follows:,“continue,” “temporary cessation,” and “complete cessation.”

### Difference between QV and UPCR in proteinuria CTCAE grade

To assess the difference between UPCR and QV in evaluating proteinuria grading during chemotherapy, we investigated all the points where UPCR and QV could be measured simultaneously. Both the UPCR and QV were assessed based on the CTCAE ver 5.0, and a UPCR of < 1.0 and a QV of 1+ were evaluated as Grade 1; a UPCR ≥1.0 and < 3.5, and a QV of 2+ and 3+ were evaluated as Grade 2; and a UPCR ≥3.5 and a QV of 4+ were evaluated as Grade 3. The concordance rate for CTCAE Grade 2 proteinuria was calculated as the rate of samples that matched the CTCAE Grade 2 evaluation to the total number of samples (*n* = 1026).

### Statistical analysis

We assumed that the ratio of the worst UPCR levels < 2.0 among the patients with ≥2+ proteinuria by a QV test will be 84.0%, which is equal to that reported in a previous non-English paper in Japanese domestic clinical study on the relationship between UPCR and QV. Consequently, the required sample size was estimated to be 105 using the Wilson score interval method, to maintain with 15% as a one-side range of 95% confidence interval (CI), with a two-sided alpha value of 0.05 and a power of 80%. Patient characteristics were summarized using descriptive statistics. Associations of different clinical items (age, sex, body weight, ECOG PS, primary site, used anti-VEGF/Ri, prior anti-VEGF/Ri usage, first observed QV status, and worst QV status) with low or high UPCR as categorical variables were analyzed using the chi-square test or Fisher’s exact test. Continuous variables, such as age and body weight, were evaluated using the Mann–Whitney U test. Fisher’s exact test was used when the frequency of any cell of the contingency table was ≤5. The analysis of factors related to high UPCR were verified using the Pearson correlation analysis, multivariable logistic regression analysis (the backward stepwise [Wald] regression), and the Youden index was calculated by receiver operating characteristic (ROC) curve analysis to decide the cutoff value for predicting high UPCR [6]. The correlation between the UPCR value and QV was evaluated using the Pearson correlation coefficient. The decision-making assessment when proteinuria worsened was evaluated by chi-square test and Fisher’s exact test for each QV and UPCR. Statistical significance was set at *P* < 0.05. Statistical calculations were performed using SPSS for Macintosh (release 24.0; SPSS Inc., Chicago, IL, USA).

## Results

### Patient characteristics

**Fig. 1:**
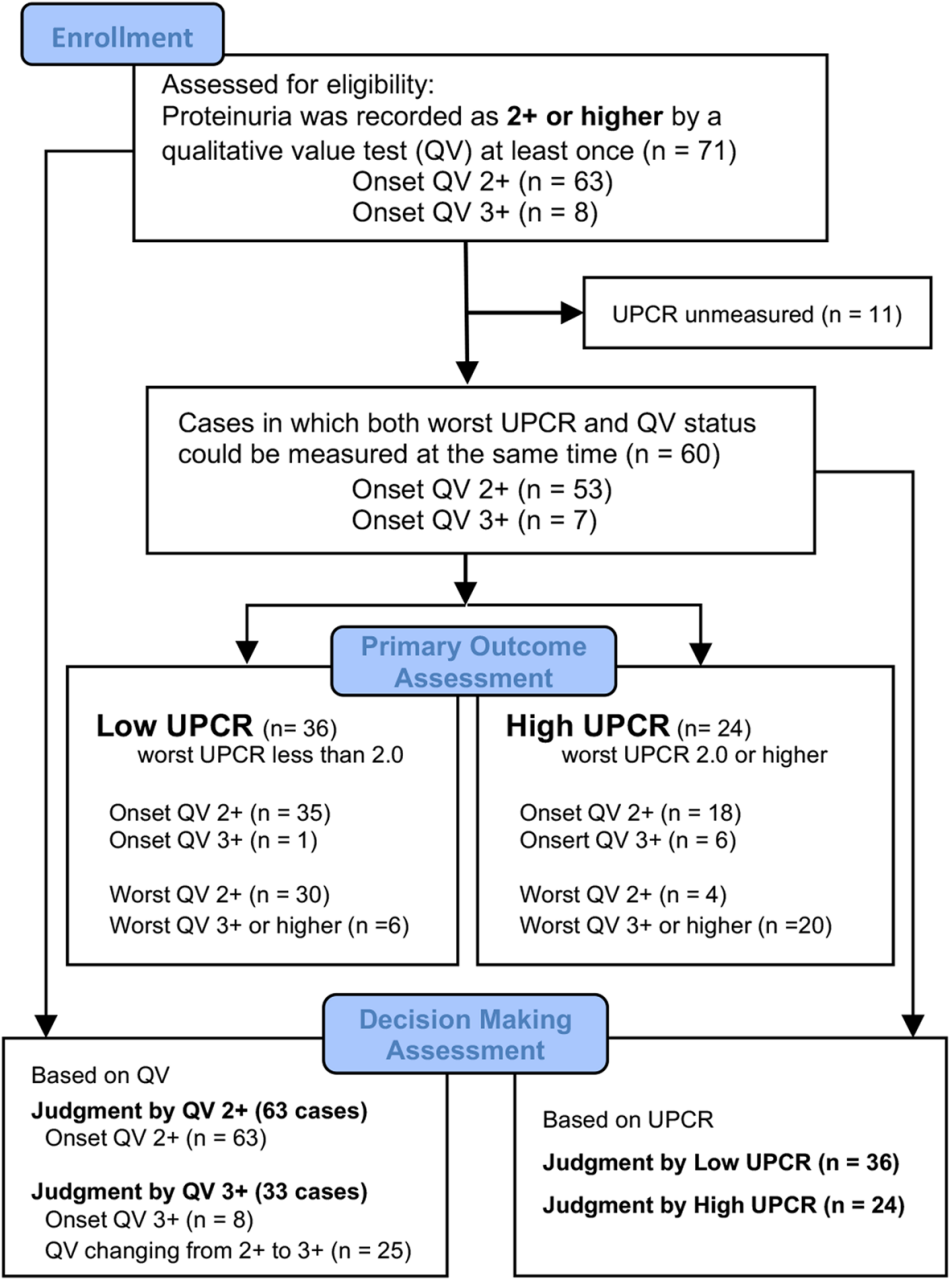
STROBE flow chart of patient enrollment

From January 2017 to December 2018, 71 patients were enrolled at seven Japanese institutions participating in the Onco-nephrology Consortium (Fig. 1). Of the 71 cases with a QV of ≥2+ at least once during the study period, 63 patients had onset QV2+, and eight patients had onset QV3+ or higher. The UPCR was not measured in 11 patients. The full analysis set (FAS) for assessing the primary outcome included 60 patients (38 men, 22 women; age, 36–86 years; median age, 67.4 years). Table 1 presents the baseline characteristics of the FAS.

**Table 1:** Baseline characteristics in the entire cohort and in patients with low and high UPCR

	Total (*n* = 60)	Low UPCR (*n* = 36)	High UPCR (*n* = 24)	P^*^
Mean age, years (range)	67.4 (36–86)	67.2 (36–86)	67.8 (46–82)	0.958
Male, n(%)	38 (63.3)	22 (61.1)	16 (66.7)	0.66
Mean body weight, Kg (range)	57.5 (32.7–101)	54.5 (32.7–81.5)	62.0 (44.4–101)	0.036
ECOG PS, n(%)				0.831
0	43 (72)	25 (69)	18 (75)	
1	11 (18)	7 (19)	4 (17)	
2	2 (3)	2 (6)	0	
unknown	4 (7)	2 (6)	2 (8)	
Primary Site				0.50
Gastric cancer	11 (18)	8 (22)	3 (13)	
Colorectal cancer	49 (82)	28 (78)	21 (88)	
Pathological status				1
tub1/tub2	47 (78)	28 (78)	19 (79)	
others	13 (22)	8 (22)	5 (21)	
Used anti-VEGF/Ri				1
Bevacizumab	41 (68)	25 (69)	16 (67)	
Ramucirumab	17 (28)	10 (28)	7 (29)	
Aflibercept	2 (3)	1 (3)	1 (4)	
RAS inhibitor usage				0.06
YES	17 (28)	7 (19)	10 (42)	
NO	43 (72)	29 (81)	14 (58)	
Prior anti-VEGF/Ri usage				0.65
YES	18 (30)	10 (28)	8 (33)	
NO	42 (70)	26 (72)	16 (67)	
Onset QV status (%)				0.013
2+	53 (88)	35 (97)	18 (75)	
3+ or more	7 (12)	1 (3)	6 (25)	
Worst QV status				< 0.001
2+	34 (57)	30 (83)	4 (17)	
3+ or more	26 (43)	6 (17)	20 (83)	

*Fisher’s exact test

*Abbreviations*: *anti-VEGF/Ri* anti-vascular endothelial growth factor (VEGF) or anti-VEGF receptor (VEGFR) inhibitors, *ECOG PS* Eastern Cooperative Oncology Group Performance Status Scale, *UPCR* a single urine protein/creatinine ratio, *QV* a qualitative value test

### Primary outcome

Regarding the primary objective of this study, the proportion of low UPCR in cases whose onset QV status was 2+ (*n* = 53) was 66% (*n* = 35) (Fig. 1). Of the onset QV2+ cases whose association between onset UPCR and onset QV status could be evaluated (*n* = 41), 40 patients (97.6%) had low UPCR onset, and only one case had an onset UPCR value of ≥2.0 (2.4%) (Supplementary Table S1).

### Comparison between low and high UPCR

In a comparison between low (*n* = 36) and high UPCR cases (*n* = 24), body weight (*P* = 0.036), onset QV status (*P* = 0.013), and worst QV status (*P* < 0.001) were significantly associated with UPCR levels (Table 1). Body weight was correlated with the worst UPCR value (*r* = 0.392, *P* = 0.002; Supplementary Fig. S1A). The area under the curve of body weight for predicting high UPCR was 0.661 (95% CI: 0.523–0.799, *P* = 0.036), and the optimal cutoff value of body weight was 52.45 kg (sensitivity: 0.792, specificity: 0.528, Youden index: 2.319; Supplementary Fig. S1B). Multivariate analysis without QV status also revealed that high body weight (≧ 52.45 kg; *P* = 0.029) was an independent predictor of high UPCR (Table 2).

**Table 2:** Backward stepwise (Wald) regression analysis

Variable	UPCR High (≥ 2) or Low (< 2)	
	OR (95% CI)	*P* value
**Step 1**		
Age **≥ 60**	3.345 (0.407–27.488)	0.261
Sex **Male**	0.584 (0.138–2.481)	0.584
ECOG **PS ≥ 1**	0.834 (0.187–3.730)	0.813
Body Weight **High (≥ 52.45 kg)**	4.415 (1.025–19.020)	0.046
Primary Site **Colorectal** vs Gastric	4.093 (0.220–76.242)	0.345
Used anti-VEGF/Ri **Bev** vs Ram/AFL	0.358 (0.032–4.054)	0.407
History of anti-VEGF/Ri **YES** or NO	0.895 (0.166–4.821)	0.897
Use of anti-hypertensive agents **YES** or NO	1.235 (0.366–4.167)	0.734
**Step 8**		
Body Weight **High (≥ 52.45 kg)**	3.825 (1.148–12.741)	0.029

*Abbreviations*: *AFL* aflibercept, *anti-VEGF/Ri* anti-vascular endothelial growth factor (VEGF) or anti-VEGF receptor (VEGFR) inhibitors, *Bev* bevacizumab, *CI* confidence interval, *ECOG PS* Eastern Cooperative Oncology Group Performance Status Scale, *OR* odds ratio, *Ram* ramucirumab, *UPCR* a single urine protein/creatinine ratio, *QV* a qualitative value test

### Decision making for treatment continuation of anti-VEGF/Ri

**Fig. 2:**
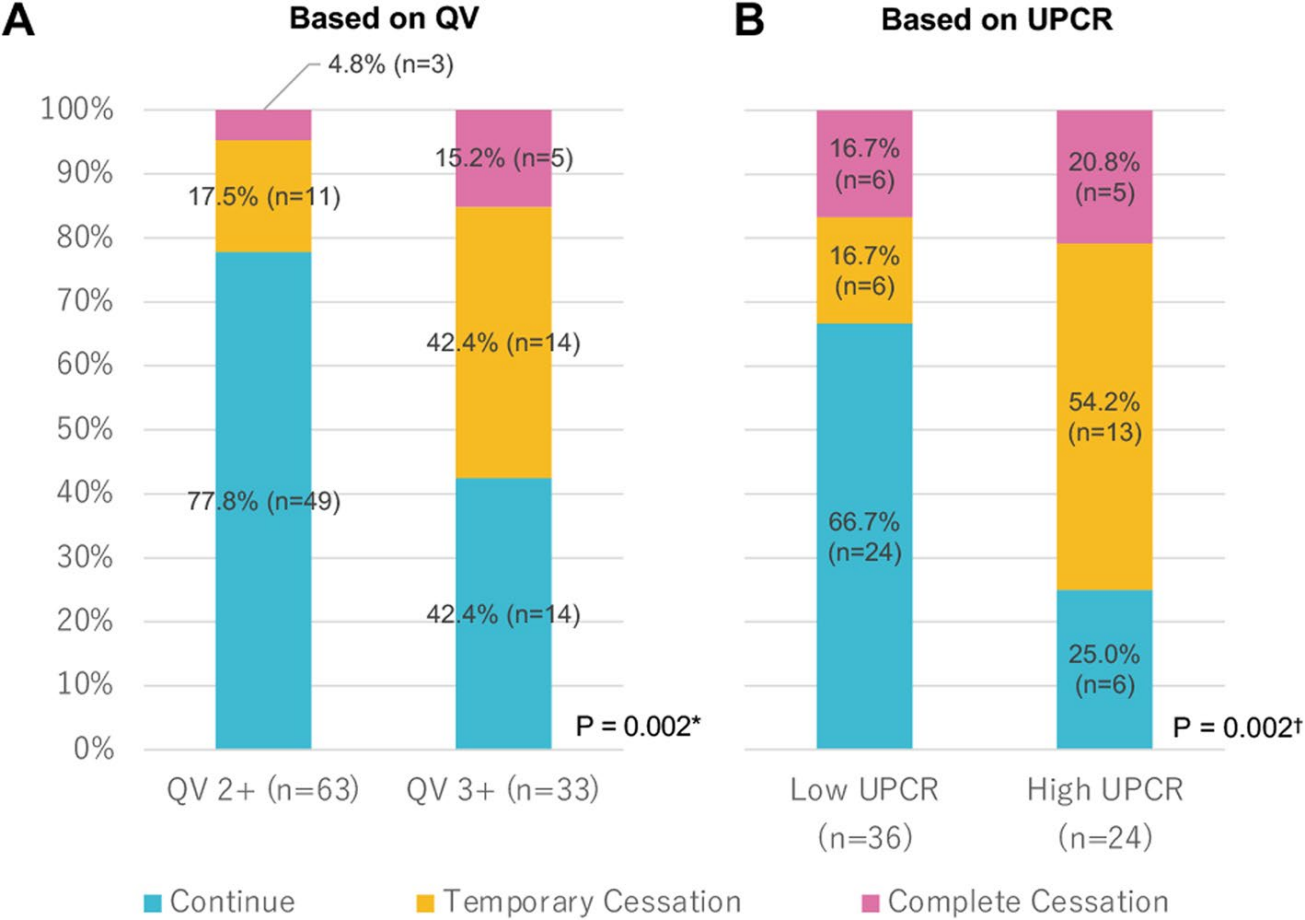
Decision making for anti-VEGF/R inhibitors continuation by QV and UPCR. Decision making for anti-VEGF/R inhibitors (**A**) based on QV and (**B**) UPCR result. *Fisher’s exact test between QV2+ and QV3+. †Fisher’s exact test between low and high UPCR. Anti-VEGF/Ri: Anti-vascular endothelial growth factor (VEGF) or anti-VEGF receptor (VEGFR) inhibitors, UPCR: Urine protein/creatinine ratio, QV: Qualitative value test

As shown in Fig. 1, the QV-based decision-making assessment was evaluated using data from 71 cases, including cases whose UPCR was not measured. The decision-making based on UPCR levels was evaluated using the data of 60 cases in which UPCR could be measured (Fig. 1). Regarding the judgment of anti-VEGF/Ri continuation based on changes in QV status, treatment was continued in 77.8% of cases when the QV became 2+, but it was only continued in 42.4% of cases when the QV became 3+ (*P* = 0.002; Fig. 2A). However, when the UPCR became high, 25.0% decided to continue treatment (*P* = 0.002; Fig. 2B), and a high UPCR was likely to be judged more carefully than a QV 3+. In addition, according to the analysis of 1026 samples that could measure UPCR and QV simultaneously, when the result of QV2 + was obtained, the decision to discontinue an anti-angiogenesis inhibitor was made at 24% (57/234) and 54% (19/35) of low and high UPCR cases, respectively (*P* < 0.001). On the other hand, when the result of QV3 + was obtained, the decision to discontinue an anti-angiogenesis inhibitor was made at 11% (5/47) and 56% (25/45) of low and high UPCR cases, respectively (*P* < 0.001, Supplementary Fig. S2).

### Correlation between UPCR value and QV status and concordance of CTCAE grading

**Fig. 3:**
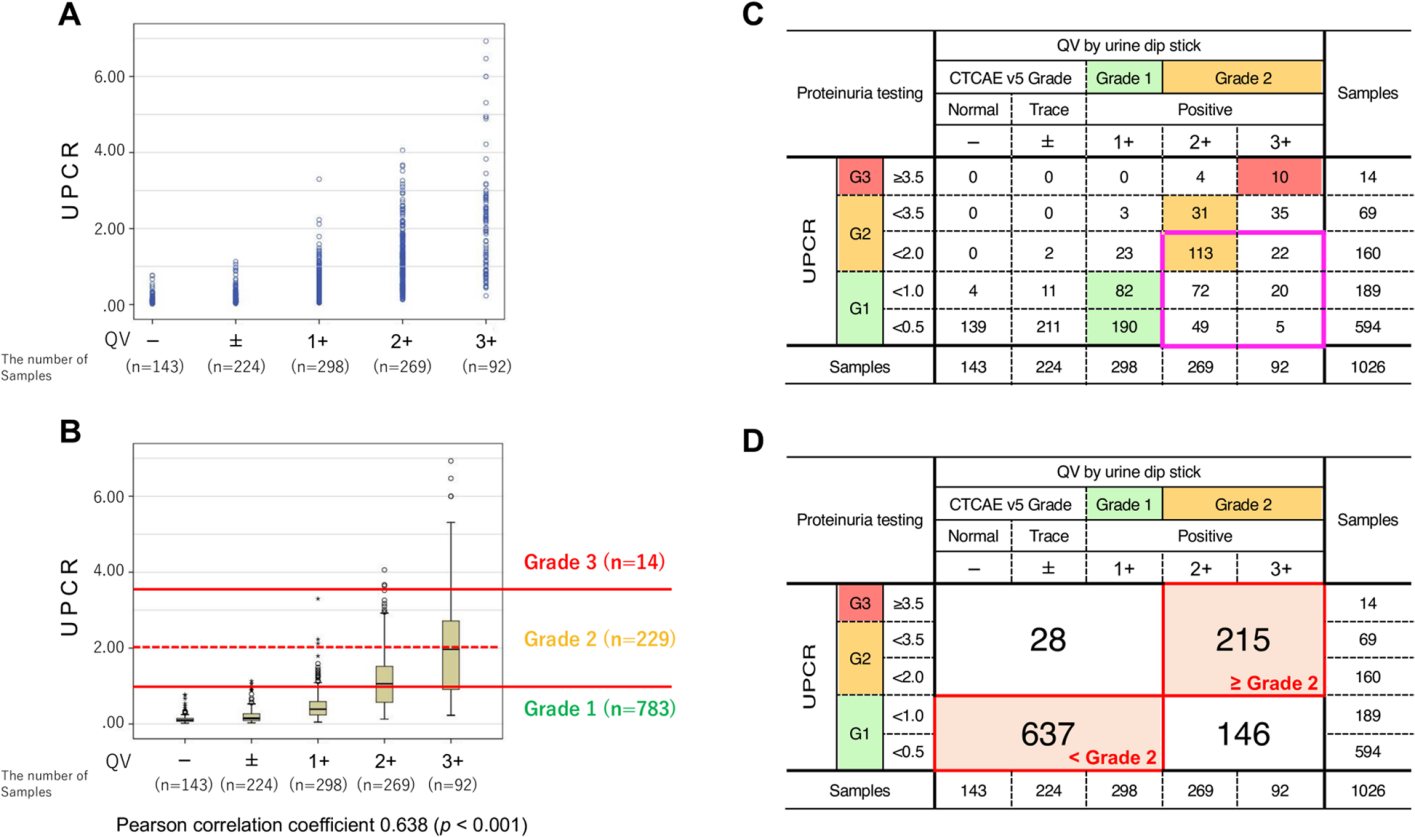
Correlation between the QV and UPCR. **A** Plot of each data sample. **B** Dashed lines indicate the cutoff value (UPCR: 2.0) customarily used to determine the postponement of anti-VEGF/Ri. **C** Green indicates Grade 1, orange indicates Grade 2, and red indicates Grade 3. The pink border shows the cases with a UPCR < 2 among the cases of QV Grade 2. **D** The red border shows that the CTCAE Grade 2 judgments are consistent in both QV and UPCR. Anti-VEGF/Ri: Anti-vascular endothelial growth factor (VEGF) or anti-VEGF receptor (VEGFR) inhibitors, CTCAE: The NCI Common Terminology Criteria for Adverse Events, UPCR: Urine protein/creatinine ratio, QV: Qualitative value test

We examined the correlation between QV status and UPCR value in 1026 samples where they could be measured simultaneously, and QV status was correlated with UPCR value (*R* = 0.638, *P* < 0.001; Fig. 3A and B). Subsequently, the relationship between the UPCR value and the CTCAE grade of QV was also evaluated. Among the 361 samples whose proteinuria CTCAE grade by QV was ≥ Grade 2, 281 samples (77.8%) were low UPCR (Fig. 3C). The concordance rate for CTCAE Grade 2 of both the QV and UPCR was 83%, and there was some discrepancy in the CTCAE grading (Fig. 3D).

### Relationship between each VEGF target agent and UPCR

We investigated the relationship between the VEGF target agents BV (*n* = 41) and Ram (*n* = 17) and UPCR, except for aflibercept (*n* = 2), which only had a small number of cases. There was a statistically significant correlation between the worst UPCR values and single doses in both BV and Ram (*r* = 0.333 [*P* = 0.033] and *r* = 0.567 [*P* = 0.018], respectively; Supplementary Figs. S3A and S4A). Ram also tended to have the worst UPCR early after the start of administration (*r* = − 0.493 [*P* = 0.045]; Supplementary Fig. S4D).

## Discussion

This study found that 66% of cases with QV2+ on qualitative tests had low UPCR (UPCR < 2.0); if the UPCR value reflects a more actual urinary proteinuria than the qualitative test result, the QV2+ result may overestimate proteinuria. The CTCAE Grade 2 concordance rate between qualitative and quantitative tests was low, and of the cases judged to be the CTCAE Grade 2 by QV, the CTCAE grade by UPCR was Grade 1 in 40% of these cases. Given the discrepancy in the CTCAE grading derived from the UPCR and QV results, unnecessary treatment interruption may occur when the judgment of continuation of treatment is made on the basis of qualitative proteinuria alone. In the current study, regarding the clinical judgment of whether to continue anti-VEGF/Ri when proteinuria occurred, the UPCR values were found to be more important than the QV status.

The cutoff value of UPCR used for proteinuria management is also an important issue. In CTCAE ver5, UPCR 1.0 and UPCR 3.5 are used as indicators to separate Grade 1 and Grade 2, and Grade 2 and Grade 3, respectively, but there is no rationale behind the use of 1.0 and 3.5 as reference values. In the JO19380 study, a phase I/II study of BV in Japanese patients with colorectal cancer, the drug suspension/discontinuation policy in the study protocol stipulated that BV could be administered if proteinuria was 2 g/24 h or less in a quantitative test by 24-h urine storage [7]. Therefore, even in actual clinical practice, a UPCR of ≤2.0 is often considered by convention as an index for resuming the administration of angiogenesis inhibitors. However, the rationale for this criterion is unclear, and its contribution to clinical outcomes is unknown. It has also been reported that when a UPCR of 2.4 is used as the cutoff value, the delineating of Grade 2 and Grade 3 proteinuria has a sensitivity of 96.9% and a specificity of 82.5% [8]. There is currently no consensus on the UPCR cutoff value that leads to clinical outcomes.

Furthermore, regarding the evaluation of proteinuria in the various clinical trials thus far, it is necessary to pay attention to which version of CTCAE is used for the evaluation. For example, the handling of QV3+ or higher was not described in CTCAE ver 4.0 and earlier versions, but in CTCAE ver 5.0, QV2+ and QV3+ were defined as Grade 2, and QV4+ was defined as Grade 3. In pivotal trials using anti-VEGF/Ri, all adverse events were evaluated with CTCAE ver 4.0 and earlier versions [1–3, 9]. Therefore, in these clinical trials, Grade 2 or higher proteinuria assessment may have been inaccurate. In addition, as shown in Fig. 3C, there were ten cases (11%) with QV3+ whose UPCR values were ≥ 3.5; therefore, a discrepancy with the UPCR value exists even then using CTCAE ver 5. Regarding proteinuria when using lenvatinib, a multikinase inhibitor with VEGFR 1–3 inhibitory activity, it has also been reported that only 36.4% of the dipstick 3+ samples were UPCR Grade 3 [10]. Therefore, CTCAE grading has its limits, and it is also necessary to pay attention to the version of CTCAE used in pivotal clinical trials.

This study showed an association between high UPCR and body weight. For BV and Ram, a single dose is prescribed based on body weight, and a single dose of each drug was found to be associated with the worst UPCR [1, 3]. Interestingly, there was no correlation between the number of doses or total dose of each drug and worst UPCR (Supplementary Figs. S3 and S4), suggesting that higher single doses carry a greater risk of proteinuria, and that a single dose rather than the total dose is a risk of proteinuria. Single doses are common in people with a high body weight, and proteinuria monitoring needs to be enhanced in these cases. In addition, if proteinuria is present, dose reduction of anti-VEGF/Ri may be effective. For example, when BV is used for colorectal cancer, 7.5 mg/kg of BV is used once every 3 weeks in combination with the capecitabine plus oxaliplatin (CapeOX) regimen, whereas 5 mg/kg is used once every 2 weeks in combination with the fluorouracil/folinic acid plus oxaliplatin (FOLFOX) regimen [1]. Considering that a single dose of BV poses a risk of proteinuria, a regimen given every 2 weeks rather than every 3 weeks may reduce the risk in cases where proteinuria is a problem. No previous clinical trial has directly compared the rate of proteinuria between BV every 2 weeks and every 3 weeks. According to the sub-analysis of the TRICOLORE study [11], a randomized phase III trial to determine whether S-1 and irinotecan plus BV is noninferior to mFOLFOX6 or CapeOX plus BV as the first-line treatment of metastatic colorectal cancer, the rate of proteinuria of Grade 3 or higher was 4 and 0% triweekly and biweekly, respectively, in the oxaliplatin-based regimen. In contrast, it was 3 and 2% triweekly and biweekly, respectively, in the irinotecan-based regimen. However, the evaluation of proteinuria may be inaccurate given that CTCAE ver 4.0 was used to evaluate adverse events in the TRICOLORE study. Still, it was suggested that the risk of proteinuria differs depending on the base regimen. In the EAGLE study [12], a randomized phase III trial comparing two doses of BV (5 mg/kg and 10 mg/kg) combined with irinotecan, 5-fluorouracil/leucovorin (FOLFIRI) in the second-line setting for metastatic colorectal cancer, there was no apparent difference in the rate of Grade 3 or higher proteinuria (1 and 0.5% in 5 mg/kg and 10 mg/kg of BV, respectively). However, the ver 3.0 of CTCAE was used to evaluate adverse events in this EAGLE study, and there was no UPCR information’ therefore, the evaluation of proteinuria was inadequate.

Regarding the administration period up to the worst UPCR, although there was no association found in the cases treated with BV, there was a tendency for worst UPCR to occur early after the start of treatment with Ram (Supplementary Figs. S3 and S4). Therefore, when using Ram, careful monitoring is required in the early stages.

There are several limitations to this study. First, this is a retrospective observational study with a small sample size. For example, although there is evidence that dietary constituents alter the risk of developing upper gastrointestinal cancers [13], these data have been obtained from observations instead of experimental studies or clinical trials, and there is a hurdle that it is hard to evaluate the actual dietary content accurately. As another example, although the microbiota has been attracting attention as a potential prognostic biomarker for gastrointestinal cancer [14], since its profile is dependent on age, diet, lifestyle, and environmental factors, further verification is needed to determine if it effectively predicts the prognosis of individual cases. Thus, in the case of retrospective observational studies, it is not suitable for investigating changes over time or searching for factors that influence the results. Similarly, in this study, although it is essential to quantify and collect changes in proteinuria for considering how changes in proteinuria affect prognosis, as the frequency of UPCR and QV measurement was not specified in advance, it was difficult to grasp the transition of proteinuria over time. Therefore, it is also difficult to calculate the cutoff value of UPCR to determine what value is acceptable for damage to the kidney, which is a future task in the Onconephrology interdisciplinary field. However, regarding the decision to continue treatment with anti-VEGF/Ri, it is possible to read from the medical records, and it was found that the UPCR results were more important in clinical practice. When examining the concordance rate between the UPCR and the QV, 1026 samples that could measure both simultaneously were used for analysis, which is a good samples size. Second, there is a lack of information on the factors that are affected by the UPCR. As mentioned above, the UPCR is affected by urine-specific gravity; however, information on urine-specific gravity and muscle mass could not be collected in this study, which is a point to be noted when conducting prospective clinical studies using UPCR in the future. Third, there is insufficient research on risk factors for proteinuria. Our results that body weight was associated with UPCR but not with history of anti-VEGF/Ri use. However, the number of cases in which anti-VEGF/Ri was used as pretreatment was as small as 30 cases, which was insufficient to examine the effects of pretreatment. Interestingly, in 32 patients who did not use anti-VEGF/Ri as a post-treatment of this study, only 5 (16%) had QV2+ proteinuria, whereas in 20 cases who used anti-VEGF/Ri as a post-treatment, proteinuria of QV2+ or higher was observed in 18 patients (90%) (Supplementary Fig. S5). That is, although the history of anti-VEGF/Ri use itself does not pose a risk of proteinuria during the continued use of anti-VEGF/Ri, in cases of Grade 2 or higher proteinuria during use of anti-VEGF/Ri, there may be a risk of proteinuria when continuing anti-VEGF/Ri. It was reported that there was no relationship between proteinuria expression and clinical outcomes in patients who used BV [15]. However, given that there is a limit to the evaluation method of proteinuria using the CTCAE grade, it is essential to consider how to best evaluate proteinuria by considering the association between proteinuria and clinical outcomes.

## Conclusions

This study found that the judgment using UPCR is more critical in determining the treatment policy than the result of the QV in the evaluation of proteinuria. In the future, When conducting clinical studies using angiogenesis inhibitors, it is also crucial to collect proteinuria data that will contribute to later discussion, leading to discussions related to renal protection or prognosis prolongation.

## Supplementary Information

**Additional file 1: Figure S1.** Correlation between body weight and UPCR. (A) Plot of each data sample. (B) ROC curve of body weight. (C) Contingency table analysis. Cross table of body weight and UPCR. *Fisher’s exact test.

**Additional file 2: Figure S2.** Difference between QV and UPCR in decision making. (A) Green indicates the cases of treatment continuation, and orange indicates the cases of treatment cessation. (B) Pink indicates QV2+/high UPCR cases, red indicates QV3+/high UPCR cases, blue indicates QV2+/low UPCR cases, and light blue indicates QV3+/low UPCR cases. *Chi-square test between QV2+/low UPCR and QV2+/high UPCR. †Fisher’s exact test between QV3+/low UPCR and QV3+/high UPCR.

**Additional file 3: Figure S3.** Correlation between worst UPCR and bevacizumab (BV). Plot data indicate the worst UPCR and (A) the BV single dose per body, (B) the total BV dose until the worst UPCR, (C) the number of BV administration, and (D) the BV treatment duration until the worst UPCR.

**Additional file 4: Figure S4.** Correlation between the worst UPCR and ramucirumab (Ram). Plot data indicate the worst UPCR and (A) the Ram single dose per body, (B) the total Ram dose until the worst UPCR, (C) the number of Ram administration, and (D) the Ram treatment duration until the worst UPCR.

**Additional file 5: Figure S5.** Proteinuria during post-treatment. (A) Percentage of anti-VEGF/Ri in subsequent treatments (*n* = 60). (B) The rate of proteinuria in patients using anti-VEGF/Ri (*n* = 20). (C) The rate of proteinuria in patients not using anti-VEGF/Ri (*n* = 32).

**Additional file 6: Table S1.** The relationship between onset QV status and onset UPCR value.

**Additional file 7: Table S2.** Associations of high proteinuria (UPCR 2 or higher) in variables.

## Data Availability

All data generated or analyzed during the current study are available from the corresponding author on reasonable request.

## Abbreviations

BV: Bevacizumab
CapeOX: Capecitabine plus oxaliplatin
CI: Confidence interval
CTCAE: Common terminology criteria for adverse events
ECOG PS: Eastern cooperative oncology group performance status scale
FAS: Full analysis set
FOLFIRI: Irinotecan, 5-fluorouracil/leucovorin
FOLFOX: Fluorouracil/folinic acid plus oxaliplatin
mTOR: Mammalian target of rapamycin
QV: Qualitative value test
Ram: Ramucirumab
ROC: Receiver operating characteristic
TKIs: Tyrosine kinase inhibitors
UMIN: The university hospital medical information network
UPCR: Urine protein/creatinine ratio
VEGF: Anti-vascular endothelial growth factor
VEGFR: Anti-VEGF receptor
VEGF/Ri: Anti-VEGF or anti-VEGFR inhibitors
