# Supplementary figures and images for: Decision making for anti-VEGF inhibitor continuation: dip stick? or urine protein/creatinine ratio? (VERSiON UP study)
Source: BMC Cancer. 2022 May 7;22:515. doi: 10.1186/s12885-022-09611-3 (PMC9080145; doi:10.1186/s12885-022-09611-3)

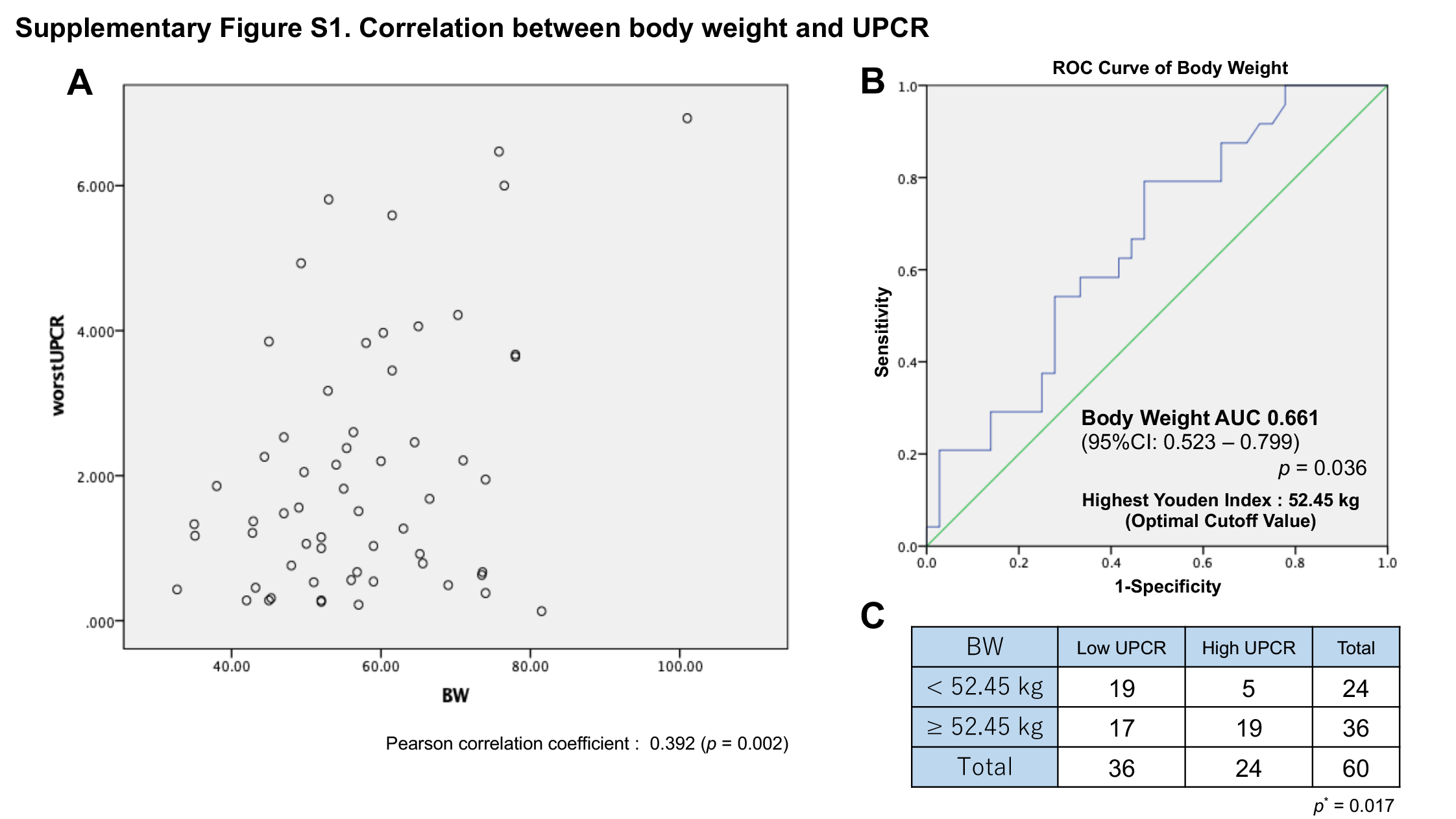

Supplement: Supplementary file 1 — Additional file 1: Figure S1. Correlation between body weight and UPCR. (A) Plot of each data sample. (B) ROC curve of body weight. (C) Contingency table analysis. Cross table of body weight and UPCR. *Fisher’s exact test. [file 12885_2022_9611_MOESM1_ESM.bmp]

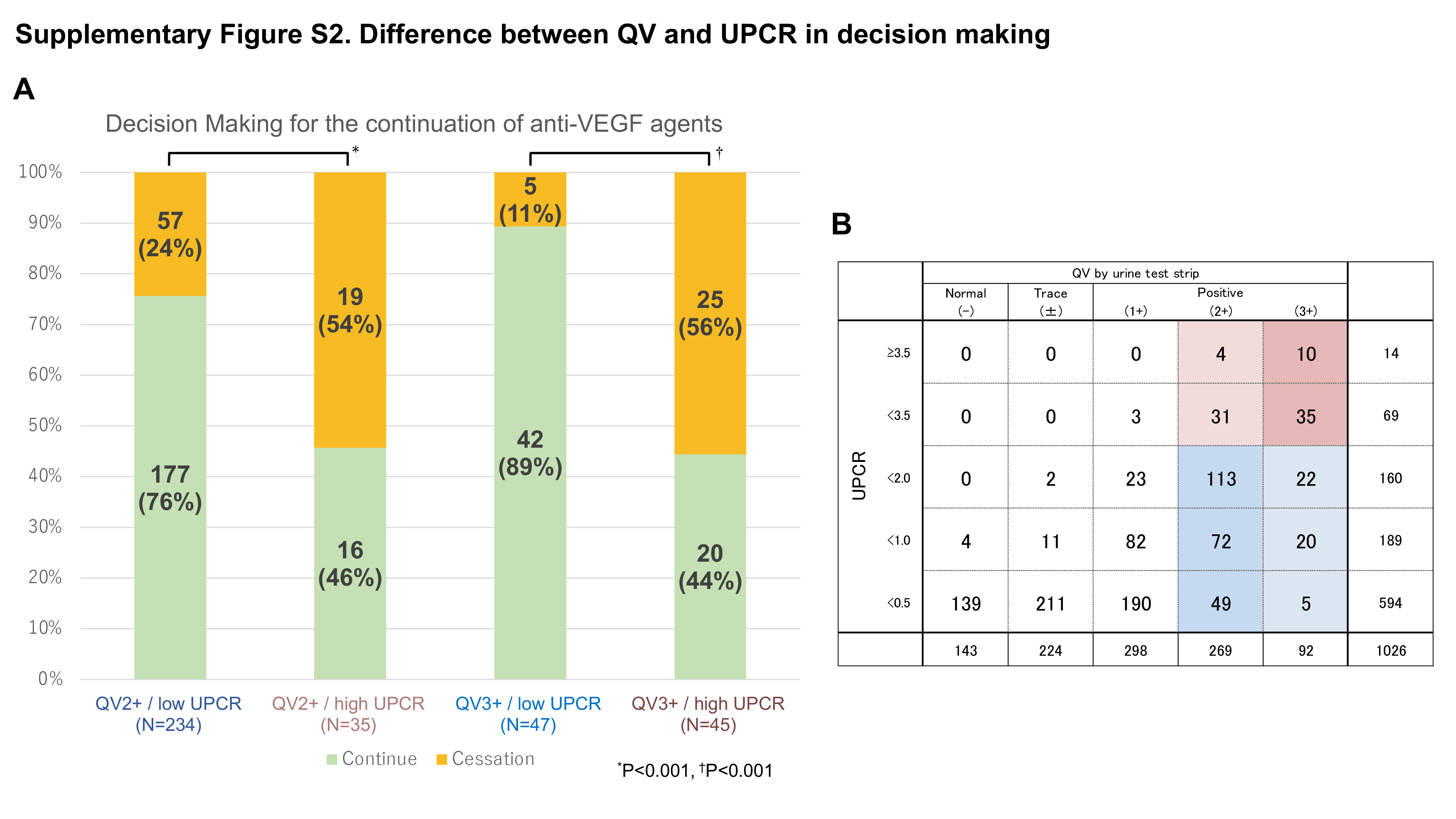

Supplement: Supplementary file 2 — Additional file 2: Figure S2. Difference between QV and UPCR in decision making. (A) Green indicates the cases of treatment continuation, and orange indicates the cases of treatment cessation. (B) Pink indicates QV2+/high UPCR cases, red indicates QV3+/high UPCR cases, blue indicates QV2+/low UPCR cases, and light blue indicates QV3+/low UPCR cases. *Chi-square test between QV2+/low UPCR and QV2+/high UPCR. †Fisher’s exact test between QV3+/low UPCR and QV3+/high UPCR. [file 12885_2022_9611_MOESM2_ESM.bmp]

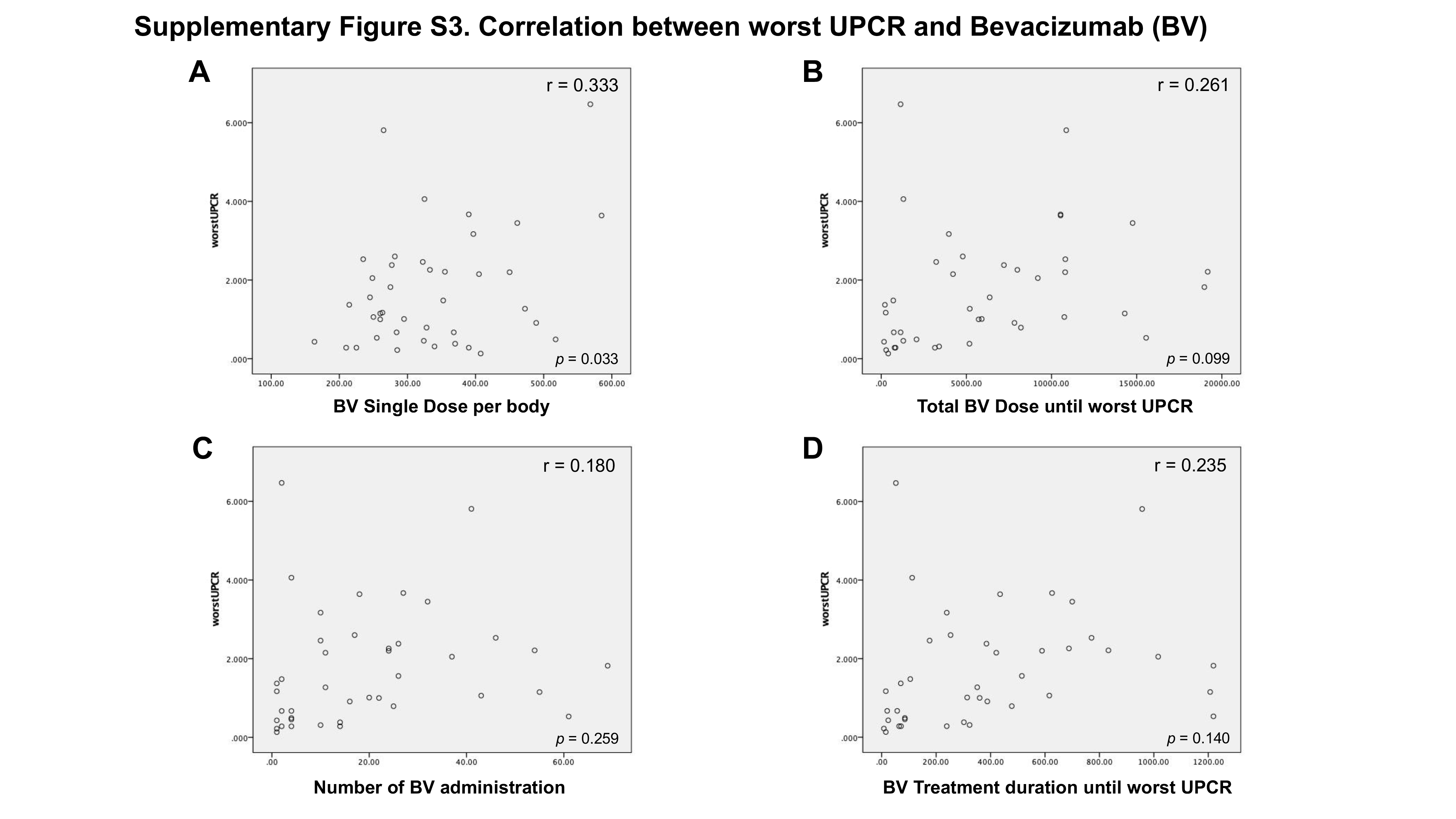

Supplement: Supplementary file 3 — Additional file 3: Figure S3. Correlation between worst UPCR and bevacizumab (BV). Plot data indicate the worst UPCR and (A) the BV single dose per body, (B) the total BV dose until the worst UPCR, (C) the number of BV administration, and (D) the BV treatment duration until the worst UPCR. [file 12885_2022_9611_MOESM3_ESM.bmp]

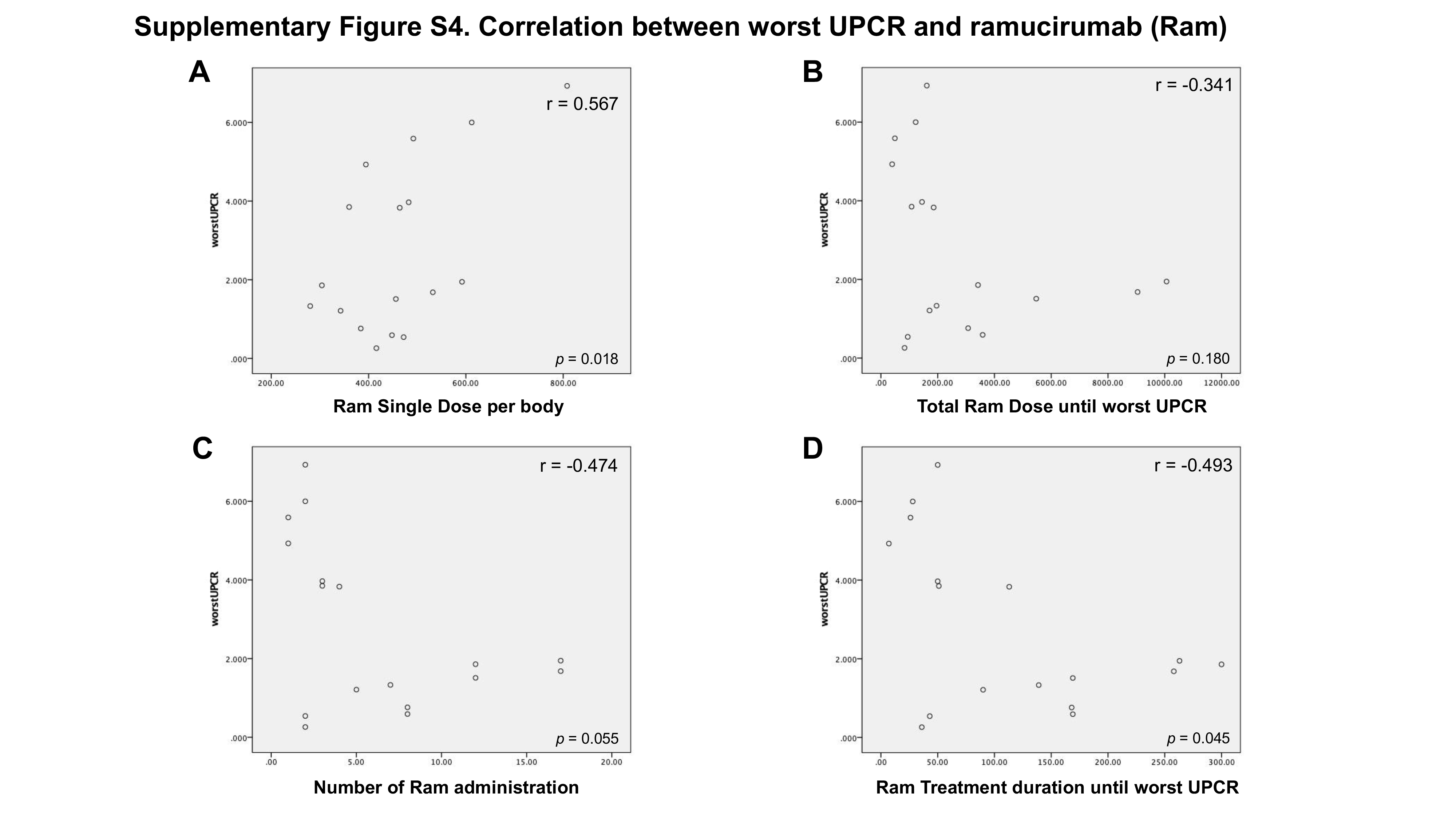

Supplement: Supplementary file 4 — Additional file 4: Figure S4. Correlation between the worst UPCR and ramucirumab (Ram). Plot data indicate the worst UPCR and (A) the Ram single dose per body, (B) the total Ram dose until the worst UPCR, (C) the number of Ram administration, and (D) the Ram treatment duration until the worst UPCR. [file 12885_2022_9611_MOESM4_ESM.bmp]

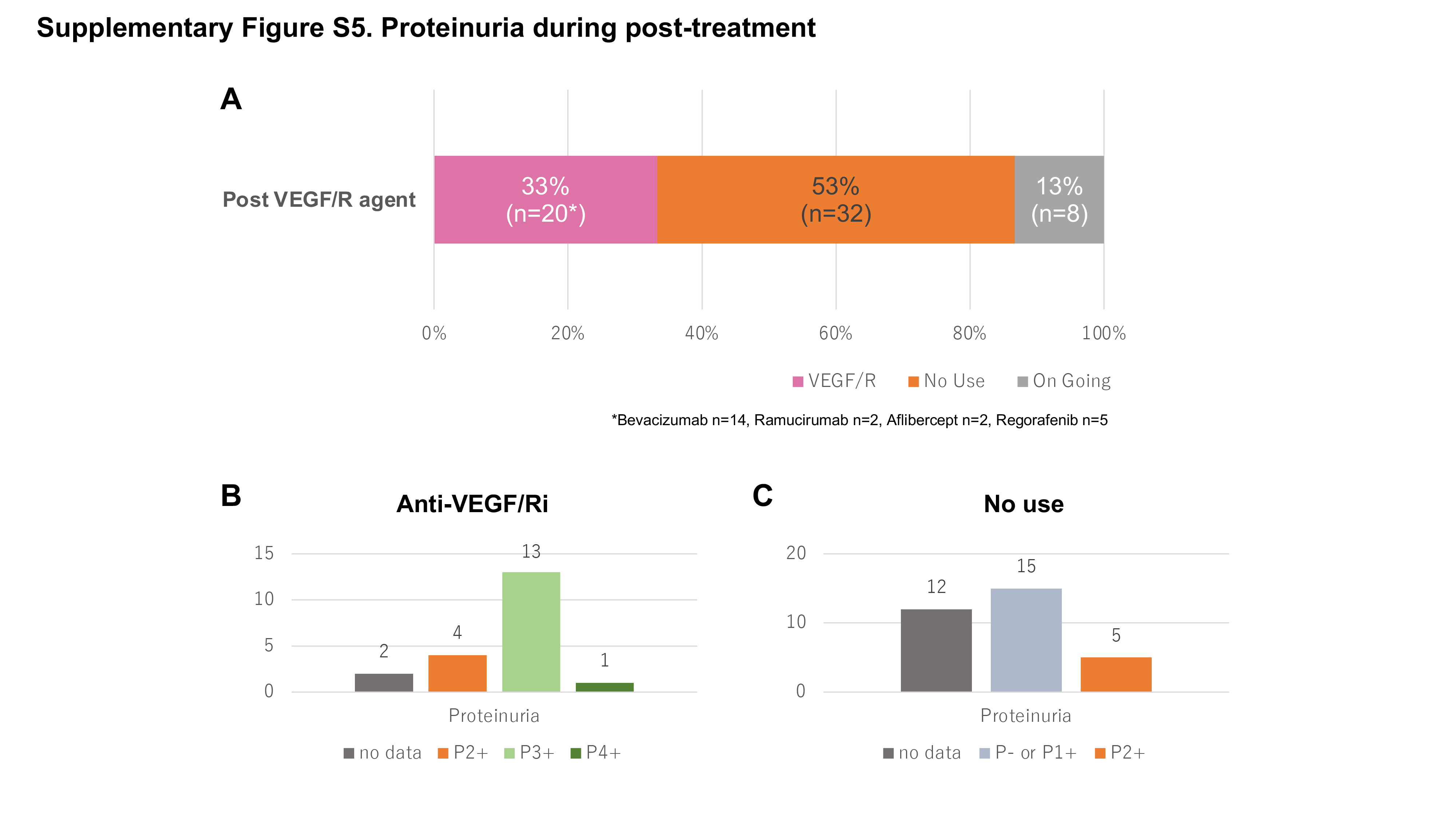

Supplement: Supplementary file 5 — Additional file 5: Figure S5. Proteinuria during post-treatment. (A) Percentage of anti-VEGF/Ri in subsequent treatments (n = 60). (B) The rate of proteinuria in patients using anti-VEGF/Ri (n = 20). (C) The rate of proteinuria in patients not using anti-VEGF/Ri (n = 32). [file 12885_2022_9611_MOESM5_ESM.bmp]
