# Supplementary material for: Decision making for anti-VEGF inhibitor continuation: dip stick? or urine protein/creatinine ratio? (VERSiON UP study)
Source: BMC Cancer. 2022 May 7;22:515. doi: 10.1186/s12885-022-09611-3 (PMC9080145; doi:10.1186/s12885-022-09611-3)
Supplement: Supplementary file 6 — Additional file 6: Table S1. The relationship between onset QV status and onset UPCR value. [file 12885_2022_9611_MOESM6_ESM.docx]

| **Supplementary Table S1.** The relationship between onset QV status and onset UPCR value | | | | | |
| --- | --- | --- | --- | --- | --- |
| **Onset status** | Total  (n = 71) | Onset UPCR less than 2.0  (n = 41) | Onset UPCR 2.0 or higher  (n = 4) | No UPCR data at QV onset  (n = 26)* | *P^†^* |
| QV 2+ (%) | 63 | 40 (97.6%) | 1 (2.4%) | 22 | 0.001 |
| QV 3+ (%) | 8 | 1 (25%) | 3 (75%) | 4 |  |
| * These data were excluded from the calculation of the percentage of UPCR in Onset QV.  † Fisher’s exact test.  Abbreviations: UPCR, a single urine protein/creatinine ratio; QV, a qualitative value test. | | | | | |
