# Supplementary material for: Decision making for anti-VEGF inhibitor continuation: dip stick? or urine protein/creatinine ratio? (VERSiON UP study)
Source: BMC Cancer. 2022 May 7;22:515. doi: 10.1186/s12885-022-09611-3 (PMC9080145; doi:10.1186/s12885-022-09611-3)
Supplement: Supplementary file 7 — Additional file 7: Table S2. Associations of high proteinuria (UPCR 2 or higher) in variables. [file 12885_2022_9611_MOESM7_ESM.docx]

| **Supplementary Table S2.** Associations of high proteinuria (UPCR 2 or higher) in variables | | |
| --- | --- | --- |
| Variable | Univariate analysis | |
|  | OR (95% CI) | *P* |
| Age **≥60** | 2.000 (0.473 – 8.462) | 0.346 |
| Sex **Male** | 1.273 (0.432 – 3.753) | 0.662 |
| ECOG **PS ≥1** | 0.617 (0.164 – 2.321) | 0.475 |
| Body Weight **High (≥52.45kg)** | 4.247 (1.302 – 13.858) | 0.017 |
| Primary Site  **Colorectal** vs Gastric | 2.000 (0.473 – 8.462) | 0.346 |
| Used anti-VEGF/Ri  **Bev** vs Ram/AFL | 0.880 (0.291 – 2.659) | 0.821 |
| History of anti VEGF/Ri  **YES** or NO | 1.300 (0.425 – 3.980) | 0.646 |
| Use of anti-hypertensive agents  **YES** or NO | 1.491 (0.520 – 4.279) | 0.457 |
| Abbreviations: AFL, aflibercept; anti-VEGF/Ri, anti-vascular endothelial growth factor (VEGF) or anti-VEGF receptor (VEGFR) inhibitors; Bev, bevacizumab; CI, confidence interval; ECOG PS, Eastern Cooperative Oncology Group Performance Status Scale; OR, odds ratio; Ram, ramucirumab; UPCR, a single urine protein/creatinine ratio; QV, a qualitative value test. | | |
